# The Cerebrovascular-Chronic Kidney Disease Connection: Perspectives and Mechanisms

**DOI:** 10.1007/s12975-016-0499-x

**Published:** 2016-09-14

**Authors:** Wei Ling Lau, Branko N. Huisa, Mark Fisher

**Affiliations:** 1 Department of Medicine, Division of Nephrology, University of California, Irvine, CA USA; 2 Department of Neurology, University of California, San Diego, CA USA; 3 Departments of Neurology, Anatomy & Neurobiology, and Pathology & Laboratory Medicine, University of California, Irvine, CA USA; 4 Department of Neurology, UC Irvine Medical Center, 101 The City Drive South, Shanbrom Hall, Room 121, Orange, CA 92868 USA

**Keywords:** Cerebrovascular disease, Blood-brain barrier, Chronic kidney disease, Microalbuminemia, Arteriolosclerosis, Arterial medial calcification, Hypertension

## Abstract

Chronic kidney disease (CKD) is an independent risk factor for the development of cerebrovascular disease, particularly small vessel disease which can manifest in a variety of phenotypes ranging from lacunes to microbleeds. Small vessel disease likely contributes to cognitive dysfunction in the CKD population. Non-traditional risk factors for vascular injury in uremia include loss of calcification inhibitors, hyperphosphatemia, increased blood pressure variability, elastinolysis, platelet dysfunction, and chronic inflammation. In this review, we discuss the putative pathways by which these mechanisms may promote cerebrovascular disease and thus increase risk of future stroke in CKD patients.

## Introduction

Cerebrovascular disease often involves small vessel disease (SVD), where complete or incomplete small subcortical lesions are associated with cognitive impairment, mood disturbances, and dementia. SVD is also the most common form of cerebrovascular pathology found in asymptomatic patients by brain imaging. There is significant heterogeneity in the clinical SVD syndromes due to both the diversity and topographical location of SVD lesions in the brain and their impact on the neuronal integrity. Cerebral changes with SVD include white matter (WM) rarefaction, cerebral microbleeds, microinfarcts, lacunar ischemic lesions, WM or global atrophy, increased perivascular spaces, and arteriolosclerosis. Most of these changes are detectable by current brain magnetic resonance imaging (MRI) techniques.

**Fig. 1:**
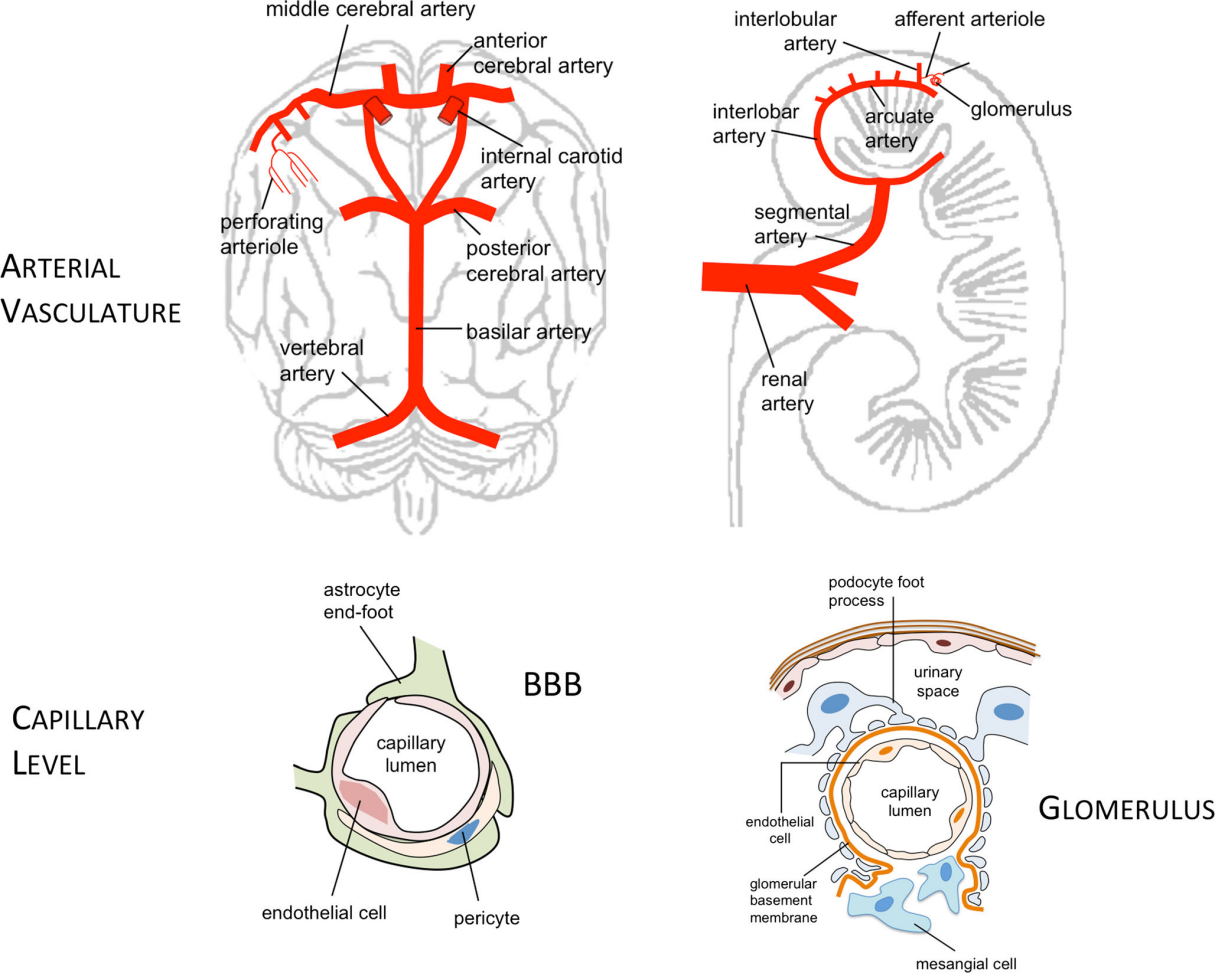
Arterial and capillary anatomy of the brain and kidney. The relatively short arterioles of the kidney and brain branch out from much larger arteries and are termed “strain arterioles”; these vessels are especially susceptible to blood pressure changes. The blood-brain-barrier (BBB) consists of the endothelial cells, the basal lamina, astrocyte foot processes, and pericytes. The human kidney contains approximately one million nephrons, each consisting of a glomerulus and renal tubule. The glomerulus is a tuft of capillary loops supported within the Bowman’s capsule by the mesangium, and consists of four cell types: the mesangial cell, glomerular endothelial cell, the podocyte (visceral epithelial cell), and the parietal epithelial cell

In the last decade, the kidney-brain interaction has garnered great interest resulting in numerous epidemiologic and mechanistic investigations. The kidney and brain share anatomical and functional characteristics making them vulnerable to similar vascular risk factors (Table 1 and Fig. 1). For example, these organs require continuous and stable high blood flow in a low vascular resistance system. They are both dependent on short, small perforating arterioles which autoregulate perfusion pressure. Both the renovascular and cerebrovascular beds are susceptible to traditional arteriosclerotic risk factors, such as aging, diabetes, hypertension, and smoking. Indeed, one could argue that declining kidney function and cognition in the elderly stem from common vascular pathogenesis. For example, hypertension is a major perpetrator of arteriosclerosis, while diabetes mellitus promotes both athero- and arteriolosclerosis. However, patients with chronic kidney disease (CKD) have a greater risk for cerebrovascular disease [1, 2] which is not explained by traditional vascular risk factors alone.

**Table 1:** Characteristics of the kidney and brain vasculature

	Kidney	Brain
Arterioles/anatomy	High pressure load per unit length	High pressure load per unit length
Arterioles/regulation	Maintenance of vascular tone	Maintenance of vascular tone
Blood flow	Constant, 360 ml/min/100 gm	Constant, 50 ml/min/100 gm
Blood barrier	Fenestrated/permeable	Tight/limited passage
Small vessels damaged by risk factors	Yes	Yes
Hypertensive pathology	Hyalinosis	Lipohyalinosis

Non-traditional CKD-related risk factors can promote cerebrovascular injury via effects on the endothelium and arterial medial wall. These factors include chronic inflammation, endothelial dysfunction, uremic toxins, anemia, and mineral-bone disorder [3, 4]. Several studies have linked CKD with different SVD phenotypes. For example, white matter hyperintensities (WMH) correlate strongly with albuminuria and decreased estimated glomerular filtration rate (eGFR) [5, 6]. WMH are areas of high signal on T2-weighted MRI that represent (at least in part) ischemia and are characterized by neuronal loss, demyelination, and gliosis—a radiological appearance often referred to as leukoaraiosis. Leukoaraiosis is significantly associated with higher risk of stroke, dementia, and death [7]. In the most advanced form of CKD, i.e., end-stage kidney disease, there is high prevalence of WMH as well as global reduction in gray matter [8, 9]. These pathologies provide a mechanistic basis for the large-scale meta-analyses that have confirmed CKD as a significant independent risk factor for stroke [10, 11].

**Fig. 2:**
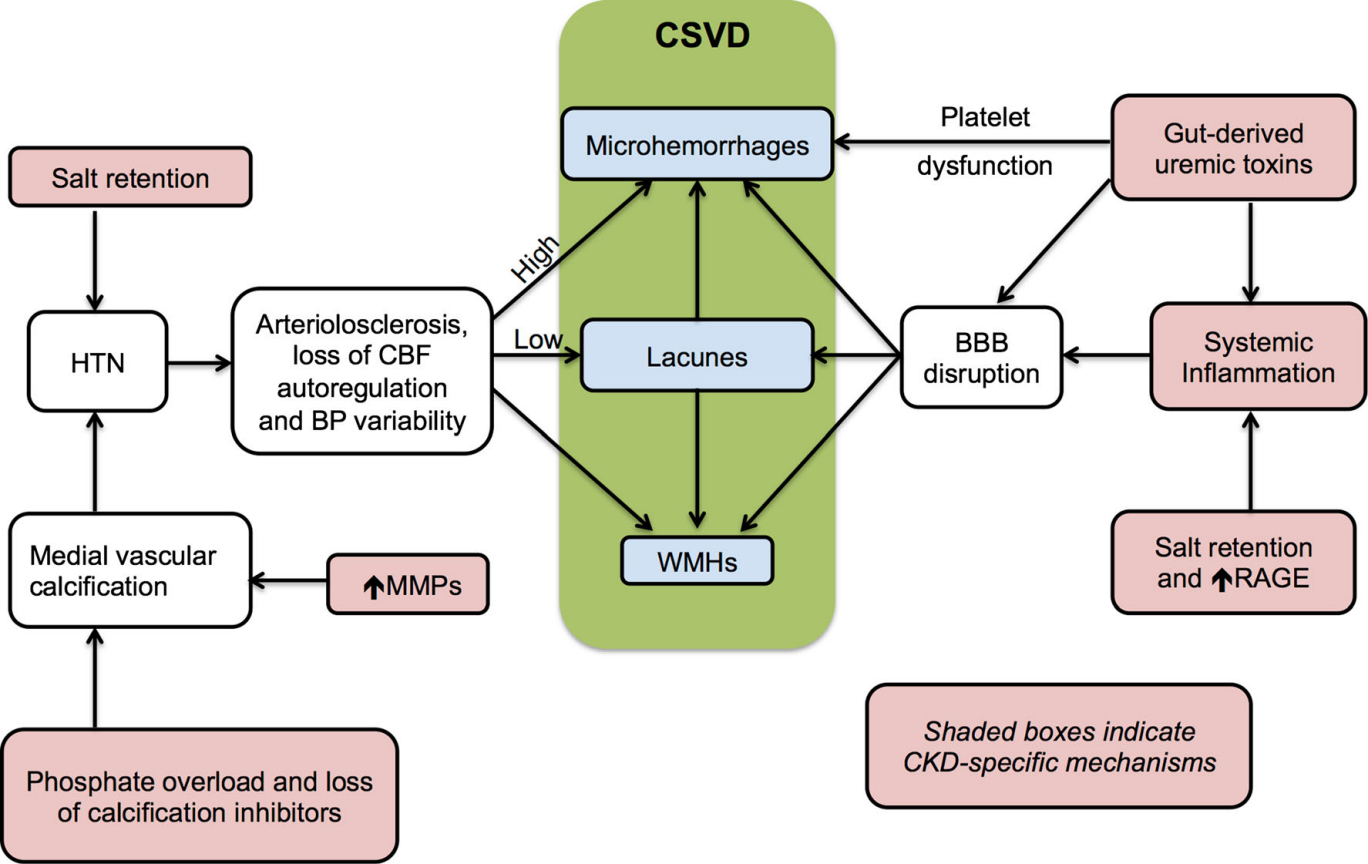
Proposed CKD-specific pathways that lead to cerebral small vessel disease (CSVD). The spectrum of CSVD ranges from white matter hyperintensities (WMHs) seen on MRI to microhemorrhages, lacunes, and microinfarcts. Hyperphosphatemia and deficiency of calcification inhibitors in the uremic milieu promote vascular calcification, hypertension (HTN), and loss of cerebral blood flow (CBF) autoregulation. Increased levels of matrix metalloproteinases (MMPs) lead to elastin degradation with subsequent increased vascular calcification. The increased blood pressure (BP) variability may be detrimental at both extremes, with high BP increasing risk of microhemorrhages and low BP predisposing to lacunes. Circulating gut-derived uremic toxins impair platelet function and drive chronic systemic inflammation, resulting in BBB endothelial dysfunction. Circulating pro-inflammatory RAGE (receptor for advanced glycation end products) ligands such as S100A12 may further promote inflammation-induced BBB disruption. CKD is a salt-avid state, and the salt overload aggravates both HTN and systemic inflammation

There is also a strong relationship between CKD and cognitive decline, and many have suggested that subclinical SVD underlies this association. In fact, patients with CKD have a typical SVD neuropsychological profile, i.e., loss of executive function and decreased processing speed [12–14]. Here, we will review unique, non-traditional factors in the uremic milieu that promote vascular injury. Further, we discuss how these factors may drive common pathways of endothelial and vascular wall damage and result in SVD phenotypes (Fig. 2). Many of these associations are currently correlative and will require confirmation in mechanistic bench and clinical studies.

## Accelerated Arteriolosclerosis with Impaired Autoregulation

The brain and kidney are similar in that both organs have high blood flow rates and have local autoregulation. On a weight per weight comparison, the kidney has twice the oxygen consumption of the brain and receives ∼20 % of cardiac output (sevenfold of brain blood flow, 360 vs. 50 ml/min × 100 g), which facilitates a high glomerular filtration rate of 100–125 ml/min [15]. The arterioles of the kidney, retina, and brain are termed “strain arterioles” as they are relatively short, branch out from much larger arteries, and are exposed to blood pressure (BP) changes (Fig. 1) [16, 17].

Autoregulation allows constant blood flow despite fluctuations in BP, to maintain cerebral perfusion pressure in the brain and GFR in the kidney. Myogenic reflexes of the smooth muscle arterioles mediate this response. Hyaline arteriolosclerosis is a common vascular lesion with aging, hypertension, and diabetes, whereby various serum proteins accumulate in the arteriolar subendothelial space (hyalinosis), often extending into the media [18]. Replacement of the arteriolar smooth muscle by hyalinoid material impairs autoregulation with subsequent transmittal of increased systemic pressure into the glomerulus and cerebral capillary beds. Loss of autoregulation also predisposes to ischemic events related to decrease of regional cerebral blood flow (CBF) [19, 20]. Hypertension worsens arteriolosclerosis, creating a vicious cycle that perpetuates end organ damage.

In the brain, a hallmark of SVD is lipohyalinosis of subcortical penetrating arteries, which is also a characteristic finding in lacunar stroke. Studies have suggested that lipohyalinosis is associated with impaired autoregulation in the brain [21]. Primary hypertensive injury in the kidney primarily affects the afferent arteriole and interlobular artery, with replacement of medial vascular smooth muscle cells (VSMC) by connective tissue [22]. Subintimal hyalinosis is often present, with signs of ischemia including glomerular and tubular atrophy and interstitial fibrosis [22]. Arteriolosclerosis with impaired autoregulation are shared injury mechanisms in the brain and kidney that may be shaped by other CKD-specific factors, as discussed below.

## Blood Pressure Variability

Long-term BP variability may be an independent risk factor for cerebral microbleeds [23]. CKD is associated with increased BP variability partly due to arterial stiffness [24]. Greater BP variability is associated with increased risk of hemorrhagic stroke in stage 3–4 CKD patients [25] and is a strong predictor of mortality in hemodialysis patients [26]. Factors that incur risk of greater variability in visit-to-visit pre-dialysis systolic BP include inadequate ultrafiltration (both excessive or inadequate volume removal can incur large BP fluctuations), activation of the renin-angiotensin-aldosterone axis, anemia, and comorbid cardiovascular disease [27].

Hemodialysis patients have impaired autonomic function as indicated by lower baroreflex sensitivity values compared with the non-dialysis population [28] and thus are less able to buffer against hemodynamic reductions during fluid removal on dialysis [29]. Myocardial stunning is also common during hemodialysis and aggravates the inability to maintain cerebral perfusion, with resultant brain ischemia [30]. Longitudinal studies have shown that reduced blood flow in normal WM predicts development of leukoaraiosis [19], and analysis of almost 3000 participants in the Rotterdam Study demonstrated that lower eGFR is independently associated with lower CBF after adjustment for cardiovascular risk factors [31]. In a UK study, brain MRI and BP variability were analyzed in hemodialysis patients [32]. Although sample size was small (∼20 patients per group), the study demonstrated development of ischemic WM changes on dialysis which were more pronounced in patients with increased intradialytic hemodynamic instability. Patients who were dialyzed at 0.5 °C below core body temperature showed improved hemodynamic stability and were protected against WM changes at 1 year [32]. Cooling of dialysate is widely used among hemodialysis clinics to promote hemodynamic stability and prevent intradialytic hypotension, and was first described in 1985 [33]. The added advantages of protective brain effects and low cost add to the appeal of this intervention in the chronic dialysis population. However, larger studies with longer follow-up periods are needed before cooled dialysate can become standard [34].

## Hyperphosphatemia and Arterial Medial Calcification

Phosphate overload occurs in late CKD due to a combination of decreased urinary phosphate excretion and continued intestinal phosphate absorption. Parathyroid disorders are common in CKD and further contribute to phosphate excess. Both hyperparathyroidism and overly suppressed parathyroid lead to decreased bone formation, preventing the skeleton from acting as a reservoir for excess calcium and phosphorus. Calcium subsequently becomes deposited at ectopic soft tissue sites including the vasculature [35, 36]. Arterial calcification and stiffness drive left ventricular hypertrophy and increase the risk for cardiovascular events and death [36].

One major mechanism by which elevated phosphate drives arterial medial calcification is via induction of osteogenic phenotype change of VSMC, whereby the VSMC cease to express SM22alpha-actin and instead express bone-forming transcription factors (Runx2, Msx2) and pro-calcification proteins (alkaline phosphatase, osteocalcin) [37, 38]. These osteogenic VSMC secrete hydroxyapatite mineral vesicles into the extracellular matrix. Evidence for in vivo VSMC phenotype change has been found in calcified vascular lesions from animals [39] and humans [40]. These phosphate-induced changes are dependent on the type III sodium-phosphate cotransporters PiT-1 and PiT-2 [41, 42]. Excess phosphate can further drives arterial medial calcification via induction of VSMC apoptosis [43].

The importance of VSMC integrity in cerebrovascular health is evident in the hereditary disorder CADASIL (cerebral autosomal dominant arteriopathy with subcortical infarcts and leukoencephalopathy) that is caused by mutations in the transmembrane receptor Notch3 [44]. Notch3 is needed for proper organization of the VSMC actin cytoskeleton [45] and has been proposed to protect VSMC from apoptosis [46]. CADASIL is a genetic form of SVD manifested as early adult onset of recurrent strokes, cognitive impairment, WMH on MRI, and generalized arteriolopathy. Gradual loss of VSMC leads to fibrosis of the tunica media in small and medium-sized penetrating arteries, reduced CBF, and subsequent lacunar infarcts and dementia [44].

## Breakdown of Medial Wall Elastin

Elevated phosphate can also perpetuate matrix mineralization via elastin degradation once osteogenic-differentiated VSMC are present [47]. Degraded elastin has increased affinity for calcium and facilitates epitactic growth of hydroxyapatite along the elastic fibers [48]. Peptides derived from elastin degradation can also drive osteogenic transformation of VSMC via binding to elastin laminin receptors on VSMC [49]. In CKD patients, increased serum levels of elastin-derived peptides are associated with higher aortic pulse-wave velocity and overall mortality risk [50]. Elastolytic enzymes are elevated in the uremic milieu and further contribute to impaired vascular function: the matrix metalloproteinases MMP-2 and MMP-9 are elevated in the arteries from CKD patients and correlate with vascular stiffness and impaired angiogenesis [51]. Elevated human brain levels of MMP-9 have been reported following acute ischemic and hemorrhagic stroke [52] and in chronic vascular dementia [53]. We postulate that chronic elastin breakdown and increased MMP activity in CKD is another pathway which promotes cerebral SVD and future stroke; this remains to be confirmed in mechanistic studies.

## Deficiency of Calcification Inhibitors

A number of endogenous factors that inhibit calcification of the arterial wall under healthy conditions are deficient in CKD. These include klotho, matrix glutamate protein (MGP), pyrophosphate, and fetuin-A [36]. MGP is normally synthesized by VSMC [54] and requires vitamin K-dependent gamma-carboxylation in order to chelate mineral and inhibit calcification [55]. Plasma levels of inactive dephosphorylated, uncarboxylated MGP (dp-ucMGP) increase progressively with CKD stage and were independently associated with severity of aortic calcification in a cohort of ∼100 CKD patients [56].

Another potent inhibitor of hydroxyapatite formation, produced by healthy VSMC, is pyrophosphate. Exogenous pyrophosphate inhibits aortic calcification in rats challenged with vitamin D3 overload [57]. Osteogenic transformation of VSMC in arterial calcification (as described above) leads to expression of alkaline phosphatase, which hydrolyzes pyrophosphate to produce inorganic phosphorus, thus promoting vascular calcification [58]. Blood pyrophosphate concentrations are low in dialysis patients [59] and correlate inversely with superficial femoral artery calcification [60].

Fetuin-A is a calcium-binding glycoprotein secreted by hepatocytes that inhibits spontaneous mineral precipitation from serum [61]. It forms stable circulating complexes containing calcium, phosphorus, and acidic proteins (calciprotein particles), which can be cleared by the liver [62]. Low serum fetuin-A correlates with cardiovascular death in dialysis patients [63, 64] and may be due to acquired deficiency of the hepatic ABCC6 transporter in CKD [65].

Klotho is a co-receptor for fibroblast growth factor-23 that mediates renal phosphate excretion via downregulation of the sodium-phosphate transporters NaPi-2a and NaPi-2c [66]. Additionally, klotho may have direct protective effects on the vascular wall via prevention of VSMC osteogenic differentiation [67]. The kidney is the major source of circulating klotho, and serum and urinary klotho levels progressively decline with CKD stages [68].

We note that while deficiency of multiple endogenous calcification inhibitors is well-documented in CKD, the impact on SVD risk is unclear at this time. For example, fetuin-A therapy in rats subjected to middle cerebral artery occlusion reduced brain infarct volume in a dose-dependent manner [69]; however, there were conflicting data from a European case-cohort study in which higher plasma fetuin-A levels were associated with increased risk of future ischemic stroke [70]. Further, the klotho gene allele KL-VS has been studied in Ashkenazi Jews and Indian cohorts and found to be associated with early onset stroke [71, 72], but mechanistic data are lacking. Further studies are needed to determine pathophysiologic pathways.

## Gut-Derived Bacterial Toxins

Breakdown of the intestinal epithelial barrier due to loss of tight junction proteins has been described in CKD animals [73, 74] and is likely responsible for the translocation of gut bacterial toxins into the systemic circulation [75, 76], thus propagating systemic inflammation and cardiovascular disease [74]. Proposed pathways for intestinal tight junction breakdown include pro-inflammatory effects of elevated urea and deficiency of the transcription factor Nrf2 [74, 77, 78]. Endotoxin (lipopolysaccharide), derived from the cell wall of Gram-negative bacteria, is measurable in the blood of dialysis patients and correlates with severity of systemic inflammation in the absence of clinically detectable infection [76]. Further, the gut microbiome is altered in CKD, leading to overgrowth of bacteria that produce uremic toxins such as indoxyl sulfate, p-cresyl sulfate, and trimethylamine-N-oxide (TMAO) [74]. These toxins correlate with systemic inflammatory markers, vascular stiffness, and increased mortality risk in CKD patients [79, 80].

Data regarding gut-brain associations and SVD are now emerging. Systemic endotoxin is used in murine experiments to study brain vascular inflammation and microbleed formation [81, 82]. A recent report in a Chinese patient cohort found that ischemic stroke and transient ischemic attack correlated with altered gut microbiome [83]. In contrast to the adverse associations reported with high levels of uremic toxins in the CKD population, Yin et al. reported that blood TMAO levels were *lower* in stroke and transient ischemic attack patients compared to control subjects with asymptomatic atherosclerosis [83]. Further studies are needed to determine the modulation by uremia on the cerebrovascular effects of gut-derived bacterial toxins.

## Salt Retention

Inflammation in CKD is further aggravated by CKD being a sodium-avid state. When the diseased kidney is unable to excrete excess sodium, BP rises to effect a pressure natriuresis [84], and this hypertension is injurious to the vasculature. Stroke-prone hypertensive rats fed a high-salt diet were noted to develop malignant hypertension, and blood brain barrier (BBB) breakdown preceded intracerebral hemorrhage by up to 2 weeks [85]. There is a potential direct effect of salt on the cerebral small vessel endothelium in addition to any hypertensive injury. Salt itself is toxic and stimulates production of reactive oxygen species and inflammatory cytokines from the kidney cortex [86, 87].

In postmortem gene expression microarray of the brains from patients with SVD, Ritz and colleagues identified upregulation of inflammation via the adipocytokine and cytokine-cytokine receptor interaction pathways [88]. In a subgroup analysis of the Framingham Heart Study correlating circulating biomarkers of inflammation with brain MRI, elevated intercellular adhesion molecule-1 was associated with greater burden of WMH [89]. Intercellular adhesion molecule-1 reflects endothelial dysfunction and has also been strongly associated with progression of urinary protein loss in diabetic nephropathy [90]. Prospective studies are needed to confirm the association of endothelial and inflammatory markers with progression of CKD and SVD.

## Blood-Brain Barrier Disruption

Tight junction complexes are critical for the microstructural integrity of both the BBB and the kidney glomerulus (Fig. 1). Neuronal-capillary interactions at the BBB involve the endothelial cells, basal lamina, astrocyte foot processes, and pericytes. Tight junctions between the endothelial cells serve to restrict the passage of solutes. The BBB is susceptible to changes in blood-flow, ischemia, and inflammatory stimuli. Disruption of neurovascular coupling in turn modulates local CBF [91, 92]. In the kidney, the glomerular capillary tuft in Bowman’s capsule consists of four cell types: the mesangial cell, glomerular endothelial cell, the podocyte (visceral epithelial cell), and the parietal epithelial cell. The highly specialized interdigitating foot processes of the podocytes form a 40 nm wide slit diaphragm that is highly permeable to water and small solutes [93]. The glomerular filtration barrier has three layers: the endothelial cell, glomerular basement membrane, and the podocyte; it produces an ultra-filtrate from plasma that is destined to be excreted as urine. The endothelium of the BBB and glomerulus share similar transmembrane and cytoplasmic anchoring proteins. The podocyte slit diaphragm contains additional specialized structural molecules such as nephrin and podocin [93].

There is increased permeability of the BBB in patients with SVD [94]. BBB disruption may play an important role in SVD, possibly through toxic effects of leaked fluid and blood-derived proteins within the WM [95]. Little is known about BBB integrity in CKD. A few animal models of acute and chronic renal failure have shown BBB disruption in the setting of uremia [96, 97] but underlying mechanisms remain unclear. The study of BBB permeability via brain MRI with contrast is relatively contraindicated in patients with CKD due to concerns of nephrogenic systemic fibrosis [98]. Nevertheless, there are a few reports showing leakage of gadolinium into the CSF in patients with CKD after contrast brain MRI [99, 100]. Extravasation of contrast from the capillary bed suggests disruption of BBB integrity in these patients.

A recent study demonstrated deleterious effects of two uremic toxins, phosphate and indoxyl sulfate, on cultured mouse brain endothelial cells [101]. Both toxins induced production of reactive oxygen species and decreased cell viability; phosphate additionally caused eNOS uncoupling [101]. Indoxyl sulfate can induce reactive oxygen species production in various cell types other than vascular endothelial cells, including VSMC, renal tubular cells, monocytes, and macrophages [102–105]. These findings remain to be validated using in vivo studies of BBB integrity. Potential BBB injury via the soluble receptor for advanced glycation end products (sRAGE) pathway is discussed in the “Cerebral Microbleeds” section below. Both *p*-cresyl sulfate and indoxyl sulfate inhibit endothelial cell proliferation and induce the release of endothelial microparticles, a marker of endothelial cell damage [106, 107]; indoxyl sulfate also induces junctional breakdown via MEK-ERK-mediated phosphorylation of the myosin light chain kinase and myosin light chain [108].

## Cerebral Microbleeds

The term “cerebral microbleeds” refers to focal areas of signal loss in brain parenchyma measuring ≤10 mm on T2*-weighted gradient-recalled echo or susceptibility-weighted MRI due to hemosiderin deposits within microhemorrhages [109, 110]. In the general population, microbleeds are associated with increasing age, hypertension, cerebral amyloid angiopathy, and worse cognitive function [111, 113]. Cerebral microbleeds are prevalent in patients with SVD, and their presence has been proposed as an imaging diagnostic clue of this condition [114]. Likewise, cerebral microbleeds are commonly found on brain MRI of CKD patients [115]. Diminished eGFR appears to be an independent risk factor for cerebral microbleeds, raising the possibility that a uremic milieu may predispose to microbleed formation [115]. In a cohort of Japanese hemodialysis patients who were stroke-free at baseline, presence of cerebral microbleeds was an independent predictor of intracerebral hemorrhage during a 5-year follow-up period [116].

In a non-CKD cohort with first ever acute lacunar stroke, higher plasma S100B and lower sRAGE were independently associated with presence and number of cerebral microbleeds, especially deep microbleeds [117]. The receptor for advanced glycation end products is a transmembrane receptor that can trigger vascular inflammation; its circulating isoform sRAGE may neutralize some of the inflammatory effects via competing for binding with circulating ligands such as S100B [118]. It was proposed that the S100B/RAGE axis might play a role in SVD affecting deep brain regions by inducing inflammatory response, BBB dysfunction, and microbleeds in acute lacunar stroke [117, 118]. While sRAGE association with microbleeds has not been examined in the CKD population, sRAGE levels are 2.4-fold higher in patients with advanced CKD, and the pro-inflammatory RAGE ligand S100A12 is fourfold higher as compared to non-CKD controls [119]. In 200 incident dialysis patients followed for ∼2 years, higher S100A12 levels correlated with inflammation and increased mortality risk [119].

Uremic platelet dysfunction is another entity that predisposes to hemorrhagic complications. Platelet dysfunction in CKD is a result of combined intrinsic platelet abnormalities and impaired interaction of platelets with the vascular wall [120]. Cytoskeletal proteins are deficient, leading to reduced platelet contractility, and there is impaired binding between the surface glycoprotein complex GPIIb/IIIa with fibrinogen on the subendothelial surface [120]. Anemia in advanced CKD can further exacerbate platelet dysfunction. Erythropoietin improves platelet function not only by improving blood cell counts, but also has direct effects via increasing the density of GPIIb/IIIa surface receptors and enhancing phosphorylation of platelet proteins [121, 122].

## Conclusions

The complex interactions between cerebrovascular disease and CKD transcend common shared vascular risk factors. Many physiological and metabolic changes that occur with CKD exacerbate cardiovascular dysfunction and propagate pathogenesis of cerebrovascular disease (Fig. 2). Arterial stiffness is the result of combined endothelial and medial wall dysfunction. Circulating uremic toxins impair endothelial (intimal wall) viability, while chronic systemic inflammation in CKD contributes to both endothelial dysfunction and medial wall calcification. The latter is further magnified by hyperphosphatemia, increased elastinolysis, and deficiency of anti-calcification factors in the uremic milieu. The arterial stiffness, superimposed on sodium-avid hypertension and impaired autonomic vasomotor regulation in advanced CKD, culminates in pronounced BP variability with increased risk for both microhemorrhages and microinfarcts. Further, at the BBB level, uremic toxin-induced endothelial injury and uremic platelet dysfunction predispose to hemorrhagic events. Thus, many factors operate in tandem to accelerate cerebrovascular pathology. Further studies are needed to identify strategic targets to arrest or mitigate progression of cerebrovascular disease in patients with CKD.
